# Frequency of and sex differences in cancer treatment-related cardiac dysfunction in trastuzumab-treated patients with salivary gland cancer: a retrospective cohort study

**DOI:** 10.1186/s40959-024-00248-8

**Published:** 2024-07-17

**Authors:** Yudai Tamura, Yuichi Tamura, Yuichiro Tada

**Affiliations:** 1 Cardiovascular Center, International University of Health and Welfare Mita Hospital, Tokyo, Japan; 2 Pulmonary Hypertension Center, International University of Health and Welfare Mita Hospital, 1-4-3 Mita, Minato-ku, Tokyo, 108-8329 Japan; 3 Department of Head and Neck Oncology and Surgery, International University of Health and Welfare Mita Hospital, Tokyo, Japan

**Keywords:** Trastuzumab, Global longitudinal strain, Cancer treatment-related cardiac dysfunction, Echocardiography, Sex difference

## Abstract

**Background:**

Trastuzumab treatment for salivary gland, gastric, and breast cancer commonly causes cancer treatment-related cardiac dysfunction (CTRCD). CTRCD incidence by sex has not been well studied.

**Methods:**

This retrospective cohort study investigated frequency of and sex differences in CTRCD in patients with salivary gland cancer treated with trastuzumab at our hospital from April 2017 to March 2022. All patients underwent echocardiography at baseline and after the first, third, and sixth trastuzumab courses. We measured changes in global and regional longitudinal strain (LS) after trastuzumab administration. CTRCD was defined by left ventricular ejection fraction (LVEF) or global LS (GLS). The results were compared by sex.

**Results:**

We recorded clinical data of 49 patients (median age [IQR], 65 [55–71] years; males [75.5%]). The median follow-up period after the sixth trastuzumab course was 120 (111–128) days. One female patient and no male patient had CTRCD defined by LVEF, and two female patients (16.7%) and seven male patients (18.9%) had CTRCD, defined by GLS. The Kaplan–Meier curves showed no significant difference in CTRCD frequency, defined by GLS (log-rank, *p* = 0.88), between female and male patients. In the univariate analysis, sex was not associated with CTRCD, defined by GLS. A significant difference in apical LS was observed between baseline and the third follow-up results of male patients.

**Conclusions:**

In this study, CTRCD incidence was not significantly different between male and female patients with salivary gland cancer treated with trastuzumab. Although most previous studies have looked at female patients with breast cancer, a male patient may be found to be at similar risk of myocardial damage.

**Supplementary Information:**

The online version contains supplementary material available at 10.1186/s40959-024-00248-8.

## Introduction

Trastuzumab, a human epidermal growth factor receptor 2 (HER2)-targeted therapy, is a major cause of cancer therapy-related cardiac dysfunction (CTRCD), with decreased left ventricular ejection fraction (LVEF) in 25% of cases and symptomatic heart failure in 0.8–4.0% of cases [1–4]. Trastuzumab has been mainly used for treating HER2-positive breast cancer and indicated for treating HER2-positive gastric cancer. However, it is less commonly used for treating advanced metastatic or recurrent gastric cancer [5] and is less often evaluated for its associated cardiotoxicity. Trastuzumab-associated cardiotoxicity has mostly been reported in patients with breast cancer and more frequently in those with a history of anthracycline use, which can cause CTRCD [6]. Contemporary radiation therapy for patients with breast cancer has been reported to have little effect on longitudinal strain or cardiac biomarkers in the short term [7, 8]. Few studies have evaluated the cardiotoxicity of trastuzumab in patients with no history of anthracycline use. Recently, the number of patients using trastuzumab without a history of anthracycline use has increased because of its reported efficacy for salivary gland cancer [9–11]. The age-standardized incidence rate for salivary gland cancer was 0.56 cases per 100,000 individuals worldwide and 0.99 in Japan [12, 13]. Furthermore, although previous reports of cardiotoxicity due to trastuzumab have only been reported in female patients, the use of trastuzumab among male patients is currently more common in clinical practice. To the best of our knowledge, no studies have been conducted among men without a history of anthracycline use or with salivary gland cancer who use trastuzumab.

In cardio-oncology, biomarkers such as cardiac troponin and brain natriuretic peptide (BNP), as well as echocardiographic parameters such as left ventricular ejection fraction (LVEF) and global longitudinal strain (GLS), have been used to screen for CTRCD. These markers are also recommended in the European Society of Cardiology (ESC) 2022 guidelines as clinical parameters to be examined at baseline and follow-up in patients undergoing cancer therapy [14]. In particular, GLS is an important marker for the early recognition of myocardial dysfunction. Moreover, regional LS has recently been reported in cardio-oncology as a useful marker [15, 16]. However, studies reporting sex differences in LS changes in patients treated with trastuzumab are lacking.

Thus, this study aimed to compare trends in LS and cardiac events between male and female trastuzumab-treated patients with salivary gland cancer without a history of anthracycline use.

## Materials and methods

### Study participants

This retrospective cohort study included trastuzumab-treated patients with salivary gland cancer with no history of anthracycline use attending International University of Health and Welfare Mita Hospital, Japan between April 2017 and March 2022. The following patients were excluded: (i) those treated with anthracycline; (ii) those without data on routine follow-up by a cardiologist; (iii) those with persistent atrial fibrillation, cardiomyopathy, or previous hospitalization due to heart failure; and (iv) those for whom measuring GLS or local LS was challenging. In our hospital, all patients treated with trastuzumab are regularly followed up by cardiologists.

### Procedure for the follow-up of patients receiving trastuzumab

**Fig. 1:**
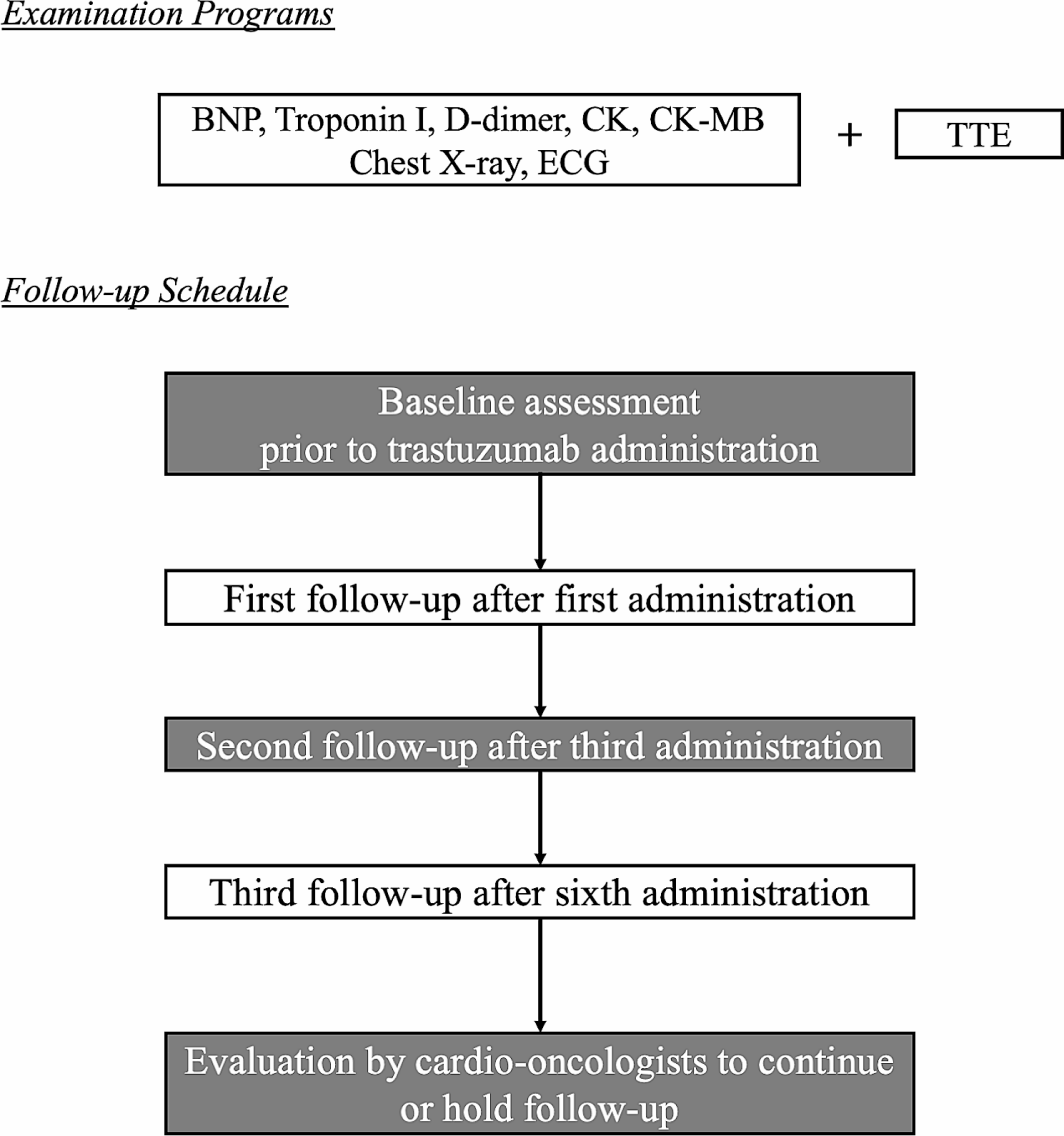
Follow-up and examinations in patients treated with trastuzumab. BNP, Brain natriuretic peptide; CK, Creatine kinase; ECG, Electrocardiogram; TTE, Transthoracic echocardiography

As shown in Fig. 1, patients who received trastuzumab were evaluated by a cardiologist before trastuzumab and after the first (first follow-up), third (second follow-up), and sixth (third follow-up) trastuzumab administrations. Follow-up after the third visit is determined by each cardio-oncologist, based primarily on echocardiographic findings. In general, patients receiving trastuzumab therapy underwent blood tests and medical, electrocardiography, echocardiography, and chest radiography examinations on the follow-up visit days. Blood tests included measurement of high-sensitivity troponin I (hsTnI), BNP, D-dimer, creatine kinase (CK), and CK-MB levels.

### Evaluation and definition of clinical variables

We collected basic characteristic data, including age, sex, body mass index, coexisting diseases, cardiac protective medications, cardiac biomarker levels, and echocardiographic parameters, from patients’ electronic medical records. Cancer-specific covariates, including cancer type and radiation therapy, were also recorded. Hypertension was defined as systolic blood pressure ≥ 140 mmHg and/or diastolic blood pressure ≥ 90 mmHg or antihypertensive medication use. Diabetes mellitus was defined as glycated hemoglobin ≥ 6.5% or receipt of insulin therapy or oral medication for diabetes mellitus. Dyslipidemia was defined as a low-density lipoprotein cholesterol level > 140 mg/dl or receipt of dyslipidemia medication. Chronic kidney disease was defined as an estimated glomerular filtration rate of < 60 mL/min/1.73 m [2].

### Echocardiographic assessment including regional LS

Experienced sonographers performed standard echocardiographic examinations according to the American Society of Echocardiography guidelines. Vivid E95 ultrasound systems (GE Healthcare, Chicago, Illinois, United States) were used, and the data were analyzed using GE EchoPAC software (GE Healthcare, Chicago, Illinois, United States).

LVEF was measured using the biplane Simpson’s method. Strain measurements were performed by a single sonographer using images obtained from apical long, four-chamber, and two-chamber views. GLS was measured automatically. Regional LS was measured using the same process and each mean peak strain value of five or six segments. Basal, mid, and apical LS values were calculated using values in the basal (six segments), mid (six segments), and apical (five segments) layers, respectively. Originally, LS was expressed as a negative value but was evaluated as an absolute value in this study. GLS and regional LS measurements were performed by an experienced sonographer and echocardiographic physician in a blinded manner.

### Definitions of clinical outcomes

We evaluated the following clinical outcomes occurring by the third follow-up visit: (i) CTRCD (LVEF), (ii) CTRCD (GLS), (iii) heart failure requiring drug intervention, (iv) discontinuation of trastuzumab owing to cardiac events, and (v) TnI elevation. For all-cause death, data were collected until the final follow-up visit. CTRCD (LVEF) was defined as a decrease of 10% compared with the baseline value and < 53% of LVEF. CTRCD (GLS) was defined as a decrease of 15% compared with the baseline value. HsTnI level elevation was defined as > 26.8 pg/mL (99th reference percentile, standard value of the Abbot hsTnI assay). If the hsTnI level at baseline was above the reference value, elevation was defined as twice the baseline level.

### Ethics approval

This study conformed to the ethical guidelines of the Declaration of Helsinki and was approved by the Ethics Committee of International University of Health and Welfare, Mita Hospital (approval number 5-21-12). The requirement for informed consent was waived due to the retrospective nature of the study.

### Statistical analysis

Continuous variables with non-normal distribution and categorical variables are presented as medians (25th–75th percentiles) and numbers (percentages), respectively. The Mann–Whitney U test was used to compare continuous variables between groups. The Wilcoxon signed-rank test was used to compare continuous variables before and after trastuzumab therapy. Fisher’s exact test was used to compare the proportions of categorical variables between the groups. Kaplan–Meier curves were plotted to determine survival from CTRCD (GLS and LVEF). Time-to-event was defined as the time from diagnosis to the occurrence of CTRCD (GLS and LVEF). The Kaplan$\:-$Meier curves generated for CTRCD-free survival were compared using the log-rank test (male or female). Hazard ratios (HRs) for the association between clinical parameters and CTRCD were analyzed using the univariate Cox proportional hazards model. The model results are presented as adjusted HRs with 95% confidence intervals (CIs). Statistical significance was set at two-sided p values < 0.05 for all tests. Statistical analyses were performed using R software version 4.1.2 (R Foundation for Statistical Computing, Vienna, Austria).

## Results

### *Baseline characteristics*

**Fig. 2:**
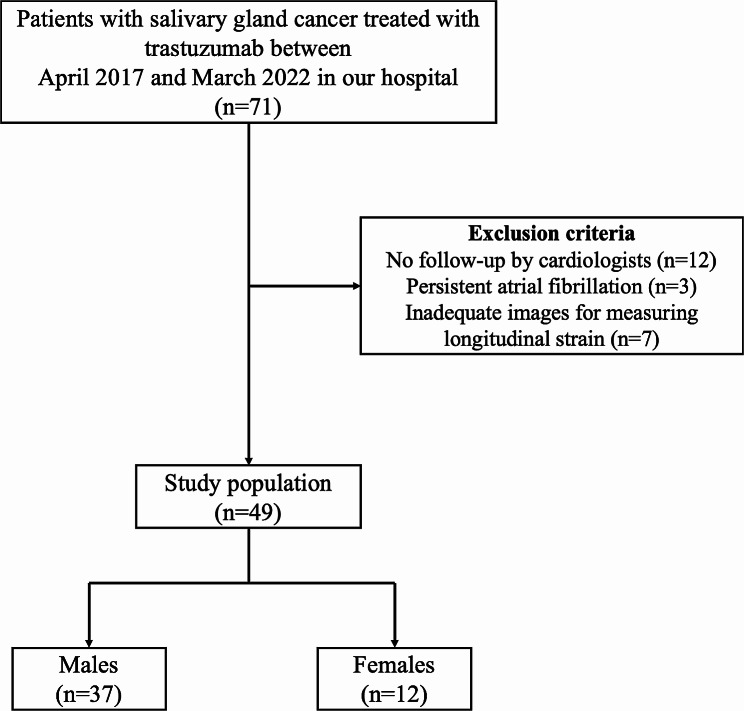
Flow diagram of the recruitment process of patients treated with trastuzumab

We enrolled 71 patients who were treated with trastuzumab for salivary gland cancer at our hospital. After excluding patients who met the exclusion criteria (Figs. 2), 49 patients (mean age [range], 65 [55–71] years; males, 75.5%) were analyzed in this study. The baseline characteristics of the study participants are presented in Table 1. The male group had significantly higher rates of hypertension (54.1% vs. 16.7%, *p* = 0.043) and renin-angiotensin system inhibitor (RASi) use (32.4% vs. 0%, *p* = 0.024) than the female group. In addition, all of the present patients were HER2-positive salivary duct carcinomas.

**Table 1:** Baseline characteristics of the study participants

	Total *n* = 49	Females *n* = 12	Males *n* = 37	*p* value
Age (years)	65 (55–71)	56 (54–67)	68 (58–72)	0.13
Body mass index (kg/m^2^)	22.4 (20.5–25.0)	21.2 (18.8–23.3)	22.9 (21.3–26.2)	0.10
**Cardiovascular risk factor and disease**				
Hypertension	22 (44.9)	2 (16.7)	20 (54.1)	0.04
Diabetes mellitus	6 (12.2)	2 (16.7)	4 (10.8)	0.63
Dyslipidemia	8 (16.3)	2 (16.7)	6 (16.2)	0.99
Chronic kidney disease	3 (6.1)	0 (0.0)	3 (8.1)	0.57
Current or prior smoking	20 (40.8)	3 (25.0)	17 (45.9)	0.31
**Baseline cardiac findings**				
BNP (pg/mL)	11.3 (5.8–22.5)	11.5 (8.1–20.4)	11.3 (5.8–22.9)	0.89
Troponin I (pg/mL)	2.5 (1.5–4.2)	1.9 (1.1–4.7)	2.5 (1.7–4.2)	0.52
LVEF (%)	67.1 (63.5–68.3)	64.8 (62.8–68.4)	67.1 (64.7–68.3)	0.36
LVEF < 50%	0 (0.0)	0 (0.0)	0 (0.0)	-
GLS (%)	18.3 (17.4–19.4)	19.3 (18.4–20.5)	18.0 (17.1–19.0)	0.02
**Pre-trastuzumab cardiac medications**				
Renin-angiotensin system inhibitor	12 (24.5)	0 (0.0)	12 (32.4)	0.02
Beta-blocker	1 (2.0)	0 (0.0)	1 (2.7)	0.99
Mineralocorticoid receptor antagonist	0 (0.0)	0 (0.0)	0 (0.0)	-
**Prior chemotherapy or radiation**				
Docetaxel (relative dose intensity) (%)	94.7 (83.4–100)	95.6 (88.9–100)	94.7 (81.8–100)	0.56
Radiation	25 (51.0)	5 (41.7)	20 (54.1)	0.73
Thoracic irradiation	1 (2.0)	1 (8.3)	0 (0.0)	0.25

Values are presented as mean ± standard deviation, median (Q1–Q3), or n (%)

BNP, Brain natriuretic peptide; GLS, Global longitudinal strain; LVEF, Left ventricular ejection fraction

### Changes in clinical parameters

The median duration by the third follow-up was 120 (111–128) days, and the median observation period by the final follow-up was 466 (293–916) days. Table 2 shows the changes in LS, LVEF, and hsTnI level by sex from baseline to the third follow-up. During the follow-up period, apical LS and GLS (*p* = 0.052) showed a decreasing trend in the male group, and a significant difference was found between baseline and the third follow-up apical LS results (*p* = 0.019).

**Table 2:** Changes in the longitudinal strain and troponin I after trastuzumab administration

	Sex	Baseline	First follow-up	Second follow-up	Third follow-up
LVEF (%)	Females	64.8 (62.8–68.4)	65.0 (62.0–68.5)	66.9 (65.1–67.1)	65.4 (63.4–68.3)
	Males	67.1 (64.7–68.3)	65.5 (64.8–67.6)	65.0 (62.6–66.9)	64.4 (62.5–65.6)
GLS (%)	Females	19.3 (18.4–20.5)	19.2 (18.2–20.6)	18.5 (17.5–19.4)	18.7 (17.4–20.7)
	Males	18.0 (17.1–19.0)	18.0 (17.2–18.8)	17.5 (16.0–18.8)	17.4 (16.3–18.1)
Relative change (%)	Females		1.5 (-6.2–4.8)	-2.9 (-10.8–1.7)	-2.3 (-6.5–4.8)
	Males		-0.6 (-6.9–5.3)	-2.8 (-7.9–5.2)	-3.0 (-7.8–2.2)
Basal LS (%)	Females	17.5 (16.8–19.2)	17.5 (16.4–18.7)	17.7 (16.9–18.6)	17.3 (16.3–18.0)
	Males	16.7 (14.8–17.5)	16.2 (15.2–17.2)	15.3 (13.8–17.7)	15.9 (14.8–17.5)
Relative change (%)	Females		-2.8 (-8.1–2.0)	-4.1 (-11.6–1.2)	0.7 (-11.4–5.3)
	Males		-1.4 (-6.5–7.3)	-1.5 (-13.4–12.4)	-1.5 (-11.7–13.4)
Mid LS (%)	Females	19.3 (18.4–20.4)	19.8 (18.5–20.9)	18.4 (17.8–19.3)	19.1 (17.1–20.1)
	Males	18.0 (17.2–19.3)	18.3 (16.8–19.3)	17.5 (16.0–19.2)	17.8 (16.7–18.8)
Relative change (%)	Females		0.5 (-2.9–4.4)	-3.2 (-7.9–1.1)	-1.5 (-9.7–7.1)
	Males		1.7 (-5.5–7.2)	-1.2 (-8.6–6.8)	0.0 (-9.5–7.6)
Apical LS (%)	Females	20.9 (20.0–23.5)	20.8 (19.6–22.4)	20.3 (19.2–23.1)	19.9 (17.5–24.4)
	Males	20.2 (18.8–22.2)	19.8 (18.2–21.8)	19.6 (17.8–21.2)	19.2 (17.7–20.1)
Relative change (%)	Females		0.0 (-13.0–12.2)	-1.3 (-8.2–2.3)	-2.9 (-13.2–6.7)
	Males		0.9 (-13.6–10.5)	-3.1 (-11.0–9.0)	-8.1 (-13.2–1.1)
Troponin I (pg/mL)	Females	1.9 (1.1–4.7)	1.4 (1.0–3.1)	2.2 (1.4–3.5)	1.4 (1.1–1.9)
	Males	2.5 (1.7–4.2)	2.7 (1.5–4.3)	2.7 (1.2–3.9)	2.4 (1.8–3.9)

Values are presented as median (Q1–Q3).

GLS, Global longitudinal strain; LS, Longitudinal strain; LVEF, Left ventricular ejection fraction

### Clinical events in patients treated with trastuzumab

The clinical outcomes of female and male patients are shown in Table 3. No significant differences in clinical events were observed between the groups. By the third follow-up, 1 and 0 CTRCD events (LVEF) occurred in the female and male groups, respectively. CTRCD (GLS) occurred in two female and seven male patients. The median time from trastuzumab initiation to CTRCD (GLS) occurrence was 55 (23–65) days. The median time to the second follow-up was 59 (55–63) days after initiating trastuzumab treatment. Four patients with CTRCD (GLS) were diagnosed at the first and second follow-ups, respectively. Details of patients with reduced GLS are presented in Supplementary Table 1.

**Table 3:** Clinical events in patients who received trastuzumab therapy

Clinical events	Total (*N* = 49)	Females (*N* = 12)	Males (*N* = 37)	*p* value
CTRCD (LVEF)	1 (2.0)	1 (8.3)	0 (0.0)	0.25
CTRCD (GLS)	9 (18.4)	2 (16.7)	7 (18.9)	0.99
Trastuzumab discontinuation due to cardiac events	1 (2.0)	1 (8.3)	0 (0.0)	0.25
Heart failure	2 (4.1)	0 (0.0)	2 (5.4)	0.99
TnI elevation	0 (0.0)	0 (0.0)	0 (0.0)	-
All-cause death	10 (20.4)	3 (25.0)	7 (18.9)	0.69

Values are presented as n (%)

CTRCD, Cancer therapy-related cardiac dysfunction; GLS, Global longitudinal strain; LS, Longitudinal strain; LVEF, Left ventricular ejection fraction; TnI, Troponin I

**Fig. 3:**
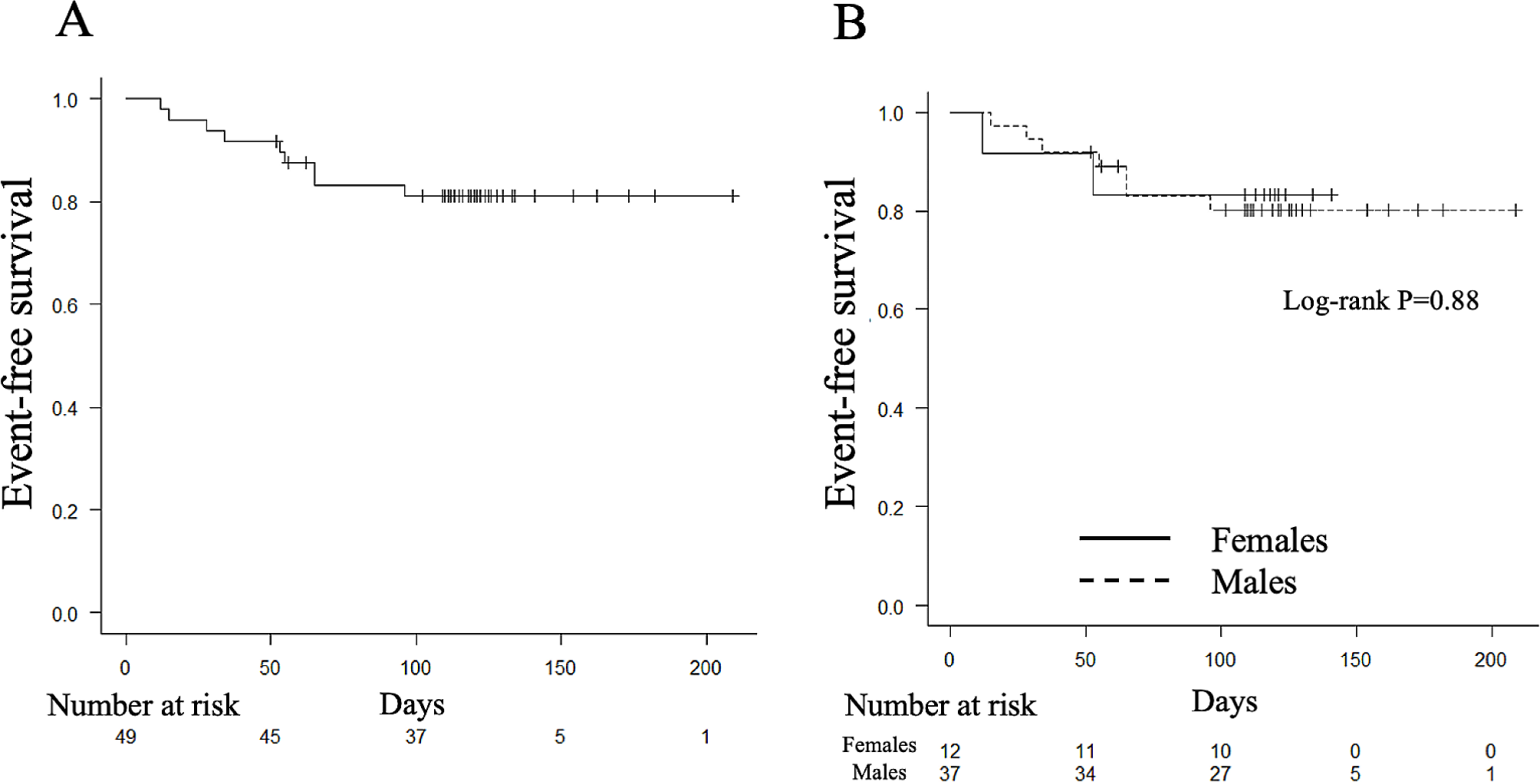
Kaplan–Meier curves of event-free survival in patients treated with trastuzumab. Event-free survival rates in patients treated with trastuzumab are presented. Events are defined as CTRCD (LVEF and GLS) incidence. Trastuzumab was administered on day 0. CTRCD, Cancer therapy-related cardiac dysfunction; GLS, Global longitudinal strain; LS, Longitudinal strain; LVEF, Left ventricular ejection fraction

All-cause death until the final follow-up occurred in three and seven patients in the female and male groups, respectively. The Kaplan–Meier curves showed that the CTRCD-free (GLS and LVEF) survival rate in patients treated with trastuzumab at 100 days was 81.1% (95% CI: 66.8–89.7; Fig. 3A), and no significant difference was found in CTRCD (GLS and LVEF) frequency between female and male patients (log-rank test, *p* = 0.88; Fig. 3B). In the univariate analysis, no factors were associated with CTRCD (GLS and LVEF) in the total cohort (Supplementary Table 2). Oral administration of RASi prior to trastuzumab initiation was also not associated with CTRCD prevention.

## Discussion

In this study, patients with salivary gland cancer who had received trastuzumab without prior anthracycline therapy were examined for trends in LS and frequency of CTRCD by sex; no significant differences were found in the frequency of CTRCD. A trend toward a decrease in apical LS with trastuzumab administration was observed in male patients.

In the present study, no significant difference was observed in the frequency of CTRCD between male and female patients. Females treated with anthracyclines have been reported to have a higher risk of CTRCD [17, 18]. However, a study using the claims database found no significant difference in the incidence of heart failure between males and females in patients treated with anti-HER2 monoclonal antibody therapy, although the majority of patients had breast cancer [19]. Even in patients with gastric cancer who probably did not receive anthracyclines, no difference in the occurrence of heart failure was observed between males and females. These findings are consistent with those of this study. Interestingly, patients receiving anthracyclines and patients receiving immune checkpoint inhibitors who had regional LS showed an early decrease in basal LS, [15, 16] whereas a trend toward a decrease in apical LS was observed in male patients treated with trastuzumab in the present study. A previous study reported that apical LS reduction occurs earlier than basal LS reduction, but the study cohort included patients with lymphoma who received anthracyclines [20]. The reason for this phenomenon could be that the blood supply to the myocardium is more easily impaired at the apical region than at the basal region. Two reasons could explain this phenomenon: first, wall shear stress due to coronary artery blood flow is lower in distal regions; [21, 22] thus, endothelial dysfunction is more likely to occur in the distal region, [23] and second, the apical region compared with the basal region has a higher workload and greater oxygen demand [24]. If this is the mechanism of cardiac dysfunction, male patients who are at a higher risk of coronary artery disease would also be at a higher risk of CTRCD. However, as the study results showed no sex differences, there may be other mechanisms of myocardial damage that are unique to trastuzumab. Therefore, at this time, we recommend that the same follow-up procedure be used for both male and female patients.

Left ventricular dysfunction occurs in up to 15–20% of patients at high to very high risk of CTRCD [25–28]. In the present study, all patients had no history of anthracycline use, heart failure, or cardiomyopathy and were aged < 80 years. Therefore, many of them are classified as having low-to-moderate risk according to the ESC 2022 guideline risk stratification for cardiotoxicity, and none are classified as having very high risk. Although the CTRCD (LVEF) incidence in this study was very low at 2.0%, CTRCD (GLS) incidence was as high as 18.4%. Trastuzumab-induced cardiac dysfunction should be considered common. Previous studies with long-term follow-up of trastuzumab-treated patients reported that 15% of patients had CTRCD defined by LVEF [29]. It has also been reported that decreased GLS in trastuzumab-treated patients is associated with decreased cardiopulmonary function as measured by peak oxygen consumption several years after cancer treatment [30]. On the other hand, overexpression of HER2 in salivary gland cancer is reported to be associated with higher disease activity and poorer prognosis. Therefore, discontinuation of trastuzumab should be avoided if possible [31, 32]. It has been reported that trastuzumab can be continued relatively safely with cardioprotective agents and close monitoring if LVEF remains at 40% or greater [33, 34]. Discontinuation of trastuzumab due to decreased GLS alone may be detrimental to the treatment of salivary gland cancer. Therefore, in patients with a relatively short-term decline in GLS, as in this study, management strategies such as the introduction of cardioprotective agents and long-term follow-up after the completion of trastuzumab may be considered. In the present study, none of the patients treated with trastuzumab alone had elevated TnI levels. This finding was consistent with that of previous reports of no elevation of TnI levels in patients without a history of anthracycline use [35, 36]. Our results also suggest that following up on GLS is important for the early identification of cardiac dysfunction.

The incidence of CTRCD in patients treated with trastuzumab was reported to be 3.2% without prior anthracycline therapy and 7–19% with prior anthracycline therapy, a difference of several folds [37, 38]. Until now, data of patients previously treated with anthracyclines have been more important because most of these patients had breast cancer. However, more patients without anthracycline use and more male patients are expected to be treated with trastuzumab in the future, and clinical data on patients treated with trastuzumab without previous anthracycline use are also important. Therefore, since CTRCD defined by GLS, even in patients treated with trastuzumab without anthracycline therapy, is relatively common, we believe that this important finding can be useful for daily clinical practice.

This study had several limitations. First, although this study conducted a retrospective analysis using a prospective screening program, the results may not be generalizable because this was a single-center study. Second, the sample size was small, and it is not certain whether we can truly establish the absence of sex differences based on the study findings. Third, a selection bias was considered because patients with poor echocardiographic quality were excluded. Finally, we could not examine long-term changes and outcomes. Therefore, further large-scale prospective studies with long-term results are required to strengthen the validity of our results.

## Conclusions

In this study, no significant difference in the incidence of CTRCD was observed between male and female patients with salivary gland cancer treated with trastuzumab and who had not previously received anthracycline. In trastuzumab-treated patients without a history of anthracycline administration, attention should be paid to the occurrence of CTRCD, irrespective of gender.

### Electronic supplementary material

Below is the link to the electronic supplementary material.

Supplementary Material 1

## Data Availability

The data that support the findings of our study are available on request from the corresponding author.

## Abbreviations

BNP: Brain natriuretic peptide
CTRCD: Cancer treatment-related cardiac dysfunction
ESC: European Society of Cardiology
GLS: Global longitudinal strain
HER2: Human epidermal growth factor receptor 2
LS: Longitudinal strain
LVEF: Left ventricular ejection fraction
