# Supplementary material for: Frequency of and sex differences in cancer treatment-related cardiac dysfunction in trastuzumab-treated patients with salivary gland cancer: a retrospective cohort study
Source: Cardiooncology. 2024 Jul 17;10:44. doi: 10.1186/s40959-024-00248-8 (PMC11253489; doi:10.1186/s40959-024-00248-8)
Supplement: Supplementary file 1 — Supplementary Material 1 [file 40959_2024_248_MOESM1_ESM.docx]

**Supplementary Table 1: Cases with reduction of global longitudinal strain or symptomatic heart failure**

| **Case** | **Sex** | **Age** | **Time of GLS decline (days)** | **Change in GLS (%)** | **Clinical course after CTRCD up to 3^rd^ follow-up** |
| --- | --- | --- | --- | --- | --- |
| 1 | Male | 58 | 96 | -24.2 | No improvements in GLS, cardioprotective drugs (-) |
| 2 | Male | 65 | 15 | -21.4 | No improvements in GLS, cardioprotective drugs (-) |
| 3 | Male | 77 | 65 | -17.2 | Improvements in GLS, cardioprotective drugs (-) |
| 4 | Male | 66 | 55 | -16.9 | Symptomatic HF (LVEF 56.2%, NYHA II) at Day 55  Improvement in GLS after cardioprotective drugs |
| 5 | Male | 76 | 65 | -19.0 | No improvements in GLS, cardioprotective drugs (-) |
| 6 | Male | 70 | 28 | -24.6 | Improvement in GLS after cardioprotective drugs |
| 7 | Male | 43 | 34 | -25.0 | No further decline in GLS after cardioprotective drugs |
| 8 | Male | 68 | none | - | Symptomatic HF (LVEF 70%, NYHA II) at Day 63  due to complete AVB |
| 9 | Female | 56 | 12 | -19.2 | Cardioprotective drugs (-) / no improvements in GLS |
| 10 | Female | 55 | 53 | -19.6 | Discontinuation of trastuzumab after decline in LVEF  Improvement in LVEF after cardioprotective drugs  No decline in GLS again after restarting trastuzumab |

In the Time of GLS decline, the date of the first administration of trastuzumab is set to day 0.

GLS, Global longitudinal strain; HF, Heart failure; LVEF, NYHA, New York Heart Association; Left ventricular ejection fraction

**Supplementary Table 2: Univariate Cox regression analysis of CTRCD**

|  |  | **Univariable** |  |
| --- | --- | --- | --- |
|  | **HR** | **95% CI** | **p value** |
| Age | 0.99 | 0.94–1.06 | 0.92 |
| Males | 1.13 | 0.23–5.43 | 0.88 |
| Hypertension | 1.45 | 0.39–5.39 | 0.58 |
| Diabetes mellitus | 0.80 | 0.10–6.42 | 0.84 |
| Dyslipidemia | 0.57 | 0.07–4.53 | 0.59 |
| Current or prior smoking | 0.70 | 0.18–2.81 | 0.62 |
| **Prior to trastuzumab administration** |  |  |  |
| RASi | 0.35 | 0.04–2.79 | 0.32 |

CI: Confidence interval, HR: Hazard ratio, GLS, Global longitudinal strain; LS, Longitudinal strain; RASi, Renin-angiotensin system inhibitor; TnI, Troponin I
